# Double Crush Syndrome as a Cause of Hemifacial Spasm

**DOI:** 10.7759/cureus.12448

**Published:** 2021-01-03

**Authors:** Jaime Guerrero, Meng Huang, Gavin Britz

**Affiliations:** 1 Neurosurgery, Houston Methodist Hospital, Houston, USA

**Keywords:** cn vii, microvascular decompression, mvd, hemifacial spasm, facial nerve, root exit zone, compression, aica, cn7

## Abstract

Hemifacial spasm is a rare but debilitating disorder of vascular compression of the facial nerve at the root exit zone causing repetitive, uncontrolled spasm of one half of the face. Usually, compression is caused by a pulsatile artery and less often by venous. Rarely, however, is hemifacial spasm caused by simultaneous compression by two formally named blood vessels. Here, we report a case of hemifacial spasm caused by simultaneous compression of the facial nerve root exit zone by the anterior inferior cerebellar artery and the superior petrosal vein. We describe the operative technique utilized to decompress the facial nerve and discuss the consequences of venous sacrifice in this scenario.

## Introduction

Hemifacial spasm (HS) is a rare disorder caused by pulsatile compression of the facial nerve root exit zone (REZ) at the pons by a vascular arterial loop. The usual offending arteries in HS include the posterior inferior cerebellar artery (PICA), anterior inferior cerebellar artery (AICA), and the vertebral artery [1-2]. Venous compression and even venous anomalies have also been reported with HS compression in HS [1,2-5]. Rarely, however, are there reports of greater than one source of compression in HS. In 1995, Janetta et al. described simultaneous venous and arterial compression in 5.9% of patients but this was by small, unnamed arteries or veins [2]. Here, we present a case of HS secondary to formally named arterial and venous double crush of the facial nerve root exit zone (REZ) and describe the operative technique utilized to relieve the compression.

## Case presentation

The patient is a 33-year-old female who presented with a two-year history of left HS that began with intermittent left eye twitching and progressed to involve the entire left side of her face. Other past medical history included hypertension and bicuspid aortic valve. She denied a family history of HS. Physical examination in the office demonstrated repetitive spasm of the orbicularis oculi. High-resolution, thin cut magnetic resonance imaging (MRI) demonstrated an obvious arterial vessel loop contacting the inferior portion of the cisternal segment of the facial nerve. Her symptoms were causing intolerable distraction at work and in her personal life, thus she ultimately opted to undergo a left-sided microvascular decompression of cranial nerve (CN) VII for relief.

**Figure 1:**
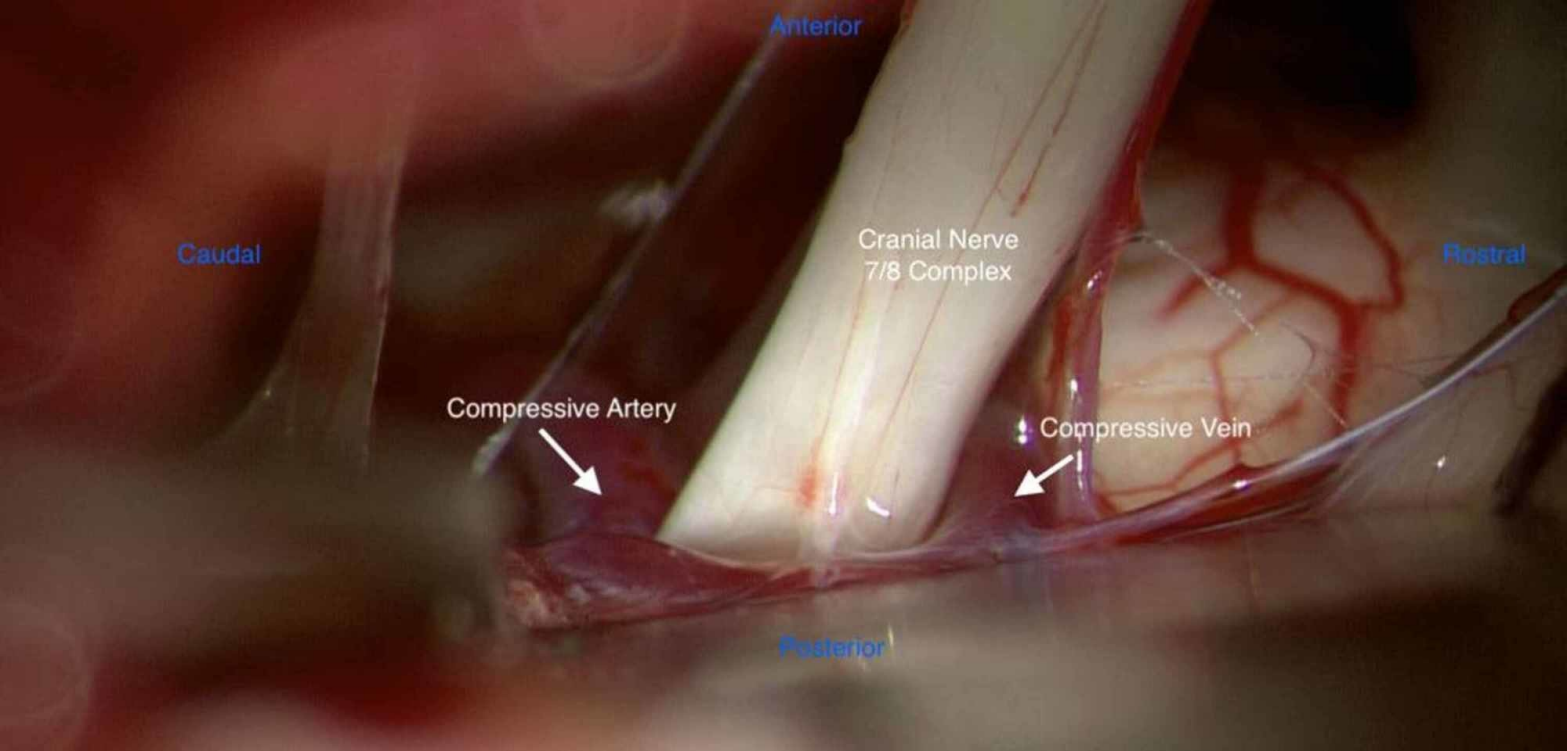
Intraoperative view demonstrating dual compression of the facial nerve root exit zone by AICA and the superior petrosal vein AICA: anterior inferior cerebellar artery

The patient underwent a small retrosigmoid craniotomy and after careful microdissection, the cerebellopontine angle and flocculonodular lobe of the cerebellum and adjacent seventh and eighth nerve complex were identified. The superior petrosal vein was identified superiorly and preserved. Careful retraction over the flocculonodular lobe of the cerebellum facilitated exposure of the facial REZ medially. A primary branch of the superior petrosal vein was then noted to be contacting the seventh nerve superiorly with AICA in direct contact with the nerve inferiorly (Figure 1).

This constituted a double crush phenomenon with a petrosal vein branch tethering the root entry zone superiorly and AICA compressing from inferiorly. We hypothesize that the venous tethering aggravated the arterial compression of the seventh nerve by preventing natural movement away from the arterial vascular loop. A standard retrosigmoid craniotomy was performed and microvascular decompression was carried out not only on the artery but also on the vein with careful dissection and mobilization. The vein was preserved as major complications have been associated with venous sacrifice. Nonresorbable Teflon patties were interposed at the nerve REZ both cranially and caudally, displacing the adjacent arterial and venous loops. Video 1 depicts the intraoperative view demonstrating careful microdissection of the offending artery and vein away from the facial nerve.

**Video 1:** Intraoperative microdissection depicting dual compression of the facial nerve by the anterior inferior cerebellar artery and a branch of the superior petrosal vein

The patient tolerated the surgery well without complications. She was discharged home on postoperative day one in stable condition. At her follow-up visit six weeks later, she endorsed the complete resolution of her HS.

## Discussion

HS is a rare condition affecting only 11 people in 100,000 in the U.S with a higher prevalence in females [1,6-7]. Similar to trigeminal neuralgia, HS is thought to be due to pulsatile vascular compression of the REZ of the facial nerve. Although there are reported cases in which other sources cause vascular compression including venous compression, to our knowledge, a “double crush” with named arterial and venous vessels has not been described in the same patient [3-4,6,8]. 

We present a patient who had a double crush of the facial nerve root exit zone (REZ) at the brainstem by the AICA, which was compressing the root entry zone inferiorly and a large venous petrosal branch tethering the root superiorly. The compressing arterial loop inferiorly was mobilized in the standard fashion and the nerve was shielded from arterial pulsations with Teflon patties. Rather than sacrificing it, the petrosal branch that was found tethering the root superiorly was also mobilized from the nerve by microsurgical dissection and shielded with Teflon patties. Venous compression in this case was largely believed to be related to tethering of the root entry zone of the seventh nerve that aggravated the arterial compression.

We took great care to preserve the petrosal vein and its primary compressive branch, as petrosal venous sacrifice can result in significant neurologic morbidity. Specifically, peduncular hallucinosis, cerebellar and even brainstem venous infarction have been reported [1,5]. A recent systematic review of 35 publications regarding the superior petrosal vein sacrifice found a 6.2% complication rate, including sigmoid thrombosis, cerebellar hemorrhage, midbrain and pontine infarct, intracerebral hematoma, cerebellar and brainstem edema, acute hydrocephalus, peduncular hallucinosis, hearing loss, facial nerve palsy, coma, and even death [5].

In a large retrospective review of 98 cases of microvascular decompression of cranial nerve five in our own institution, four patients developed clinically significant sequela of posterior fossa venous congestion evidenced by radiographic ischemia or congestive venous hemorrhagic stroke. These patients all had superior petrosal vein sacrifice. None of these complications were found in the cohort that had superior petrosal vein preservation [6].

Although the incidence is low, the neurologic morbidity associated with posterior fossa venous congestion from petrosal vein sacrifice can be severe. We, therefore, believe that petrosal vein preservation should be judiciously exercised. In our case of the double crush phenomenon in HS, we followed this principle and meticulously dissected the vein away from the nerve REZ rather than sacrificing it.

## Conclusions

Hemifacial spasm is a rare but debilitating disorder of vascular compression of the facial nerve at the root exit zone causing repetitive, uncontrolled spasm of one half of the face. Usually, compression is caused by a pulsatile artery and less often by venous. Rarely, however, is hemifacial spasm caused by simultaneous compression by two formally named blood vessels. Here, we identify a case of hemifacial spasm caused by simultaneous compression of the facial nerve root exit zone by the anterior inferior cerebellar artery and the superior petrosal vein. We describe the operative technique utilized to decompress the facial nerve and discuss the consequences of venous sacrifice in this scenario.
